# Global nucleosome occupancy in yeast

**DOI:** 10.1186/gb-2004-5-9-r62

**Published:** 2004-08-20

**Authors:** Bradley E Bernstein, Chih Long Liu, Emily L Humphrey, Ethan O Perlstein, Stuart L Schreiber

**Affiliations:** 1 Department of Chemistry and Chemical Biology, Bauer Center for Genomics Research, and Howard Hughes Medical Institute, Harvard University, 12 Oxford Street, Cambridge, MA 02138, USA; 2 Department of Pathology, Brigham and Women's Hospital and Harvard Medical School, Boston, MA 02115, USA; 3 Biological and Biomedical Sciences, Division of Medical Sciences, Harvard Medical School, Boston, MA 02115, USA; 4 Department of Molecular and Cellular Biology, Harvard University, Cambridge, MA 02138, USA

## Abstract

**Background:**

Although eukaryotic genomes are generally thought to be entirely chromatin-associated, the activated *PHO5 *promoter in yeast is largely devoid of nucleosomes. We systematically evaluated nucleosome occupancy in yeast promoters by immunoprecipitating nucleosomal DNA and quantifying enrichment by microarrays.

**Results:**

Nucleosome depletion is observed in promoters that regulate active genes and/or contain multiple evolutionarily conserved motifs that recruit transcription factors. The Rap1 consensus was the only binding motif identified in a completely unbiased search of nucleosome-depleted promoters. Nucleosome depletion in the vicinity of Rap1 consensus sites in ribosomal protein gene promoters was also observed by real-time PCR and micrococcal nuclease digestion. Nucleosome occupancy in these regions was increased by the small molecule rapamycin or, in the case of the *RPS11B *promoter, by removing the Rap1 consensus sites.

**Conclusions:**

The presence of transcription factor-binding motifs is an important determinant of nucleosome depletion. Most motifs are associated with marked depletion only when they appear in combination, consistent with a model in which transcription factors act collaboratively to exclude nucleosomes and gain access to target sites in the DNA. In contrast, Rap1-binding sites cause marked depletion under steady-state conditions. We speculate that nucleosome depletion enables Rap1 to define chromatin domains and alter them in response to environmental cues.

## Abstract 2

A genome-wide study of nucleosome occupancy at yeast promoters shows that promoters that regulate active genes, contain multiple conserved motifs, or contain Rap1 binding sites tend to be depleted of nucleosomes.

## Background

Global gene-expression patterns are established and maintained by the concerted actions of transcription factors and the proteins that constitute chromatin. The global network of interactions between transcription factors and promoters in yeast is increasingly being characterized [1]. The role of chromatin in gene regulation is less clear, however. For example, the distribution of nucleosomes, the fundamental units of chromatin, is poorly understood on a gene-specific basis, much less a global basis [2].

The nucleosome consists of approximately 146 base-pairs (bp) of DNA wrapped around an octamer of histone proteins - two each of histones H2A, H2B, H3 and H4. Eukaryotic genomes are packaged into repeating units of nucleosomes separated by around 10-80 bp of linker DNA. High occupancy by nucleosomes is thought to be generally repressive [3], and extensive remodeling (and loss) of nucleosomes occurs in the promoters of genes undergoing activation [4]. In the case of the *PHO5 *promoter in yeast, this remodeling proceeds until essentially no nucleosomes are detected across a region of several hundred base-pairs [5,6].

Transcription factors and chromatin proteins each form complex regulatory networks that interact in a variety of ways [1,7]. Transcription factors modify chromatin structure by recruiting enzymes that remodel nucleosomes or posttranslationally modify histones (by acetylation or methylation, for example) [8-10]. The modifications can be maintained through cell division and propagated to proximal nucleosomes by positive-feedback mechanisms [7,11,12]. Hence, a signal such as the activation of a transcription factor can be temporally and spatially transmitted through chromatin. Conversely, chromatin can influence transcription factor function by modulating the accessibility of target binding sites in the DNA [13,14].

We used chromatin immunoprecipitation (ChIP) and DNA microarrays to evaluate nucleosome occupancy levels for essentially all promoters in yeast. Promoters that regulate active genes, contain multiple conserved motifs or recruit Rap1 tend to be relatively nucleosome-depleted. We also used real-time PCR and micrococcal nuclease digestion to show that nucleosomes are depleted in the vicinity of Rap1 consensus sites. This depletion can be partially reversed by the actions of the small molecule rapamycin or by removing Rap1-binding sites. We suggest that other transcription factors have less robust nucleosome-depleting activities than Rap1 and must therefore act collaboratively to gain access to their cognate sites in the DNA.

## Results

### ChIP-based assay for nucleosome occupancy

**Figure 1:**
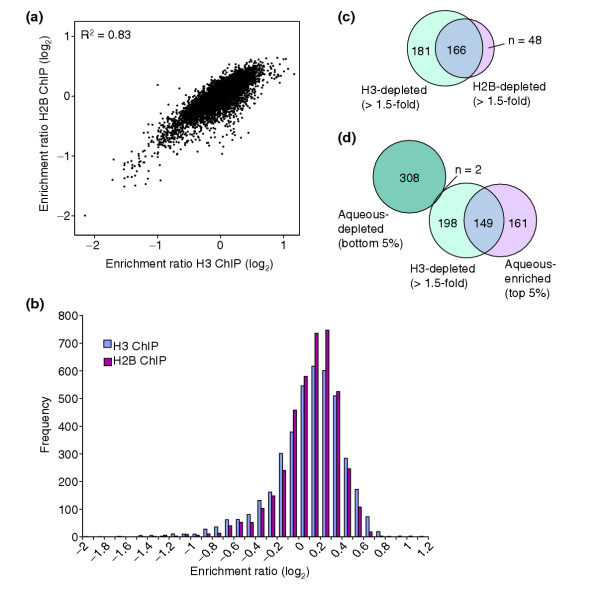
Correlation between H3 and FLAG-H2B ChIP datasets. DNA associated with histones *in vivo *was enriched in ChIP assays using antibodies against histone H3 or FLAG-H2B, and quantified by microarrays. **(a) **Relative enrichment of promoters and other non-coding regions in the H3 and H2B ChIP assays is shown. **(b) **Histogram showing distributions of enrichment for promoter regions in the H3 and H2B ChIP assays. **(c) **Overlap between regions depleted in the H3 and FLAG-H2B assays is shown. Overall, there is an 0.83 correlation between these ChIP datasets. **(d) **Overlap between regions depleted in the H3 ChIP assay and regions enriched by aqueous extraction is shown [62].

Histones are essential components of the nucleosome and efficiently cross-link to nucleosomal DNA. Antibodies against invariant portions of histones have been used previously in ChIP assays to follow nucleosome loss at the yeast *PHO5 *promoter [5,6]. We extended this approach to evaluate relative nucleosome occupancy at essentially all promoters and other intergenic regions in yeast. DNA associated *in vivo *with histone H3 was isolated by ChIP using antibody against the carboxy terminus of histone H3 (no posttranslational modifications are thought to occur in this region). ChIP DNA and unenriched control DNA were amplified by *in vitro *transcription and evaluated using microarrays. DNA associated with histone H2B was evaluated in a similar fashion using anti-FLAG antibody and a FLAG-H2B strain. H3 and H2B datasets were compiled by averaging four and three independent biological experiments, respectively. These datasets are remarkably similar as shown by a genome-wide correlation of 0.83 (Figure 1a-c). This correlation is comparable to that observed when comparing replicate H3 datasets (or H2B datasets), and suggests that both assays measure similar phenomena. In the H3 and H2B datasets, respectively, there are 347 and 214 regions depleted at least 1.5-fold relative to the average over all intergenics. In contrast, there are just 84 and 6 regions in the respective datasets enriched at least 1.5-fold relative to this average. The relatively narrow range of ChIP enrichment and the negative skew of the data (Figure 1b) are consistent with the conventional view that the majority of the genome is packaged into nucleosomes with intervening stretches of free DNA such as the activated *PHO5 *promoter [5,6].

Despite these consistencies, a possible caveat to using ChIP to evaluate nucleosome occupancy is that immunoprecipitation efficiency can depend on epitope accessibility. Rather than having low occupancies, genomic regions depleted in the H3 ChIP might be inaccessible as a result of association with large protein complexes in chromatin. To investigate this possibility, we examined a published chromatin fractionation dataset in which cross-linked chromatin fragments were subjected to phenol-chloroform extraction and DNA that partitioned into the aqueous phase was quantified by microarrays [15]. Given the polar nature of DNA and the hydrophobic nature of denatured protein, aqueous extraction should generally enrich for free DNA. We found that regions depleted in the H3 ChIP assay overlap extensively with regions enriched by aqueous extraction, but not with regions depleted by aqueous extraction (Figure 1d). Overall, there is a negative correlation of -0.54 between the H3 ChIP and aqueous-extraction datasets. Although the fractionation data may partially reflect differential cross-linking of lysines in the histone tails [15], this analysis suggests that regions depleted in the H3 ChIP experiment are relatively protein-free, as would be expected of non-nucleosomal DNA.

### Nucleosome occupancy correlates inversely with promoter strength

**Figure 2:**
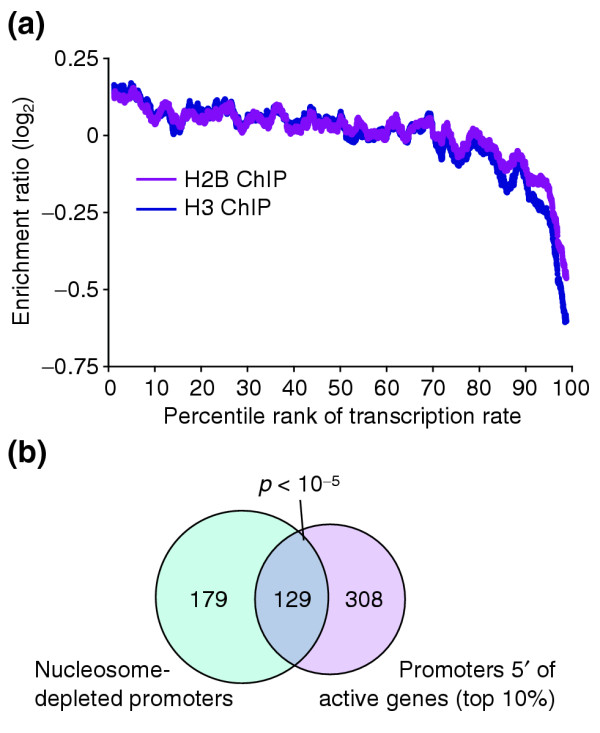
Inverse association between nucleosome occupancy and promoter strength. **(a) **Relative enrichment of promoter regions in the H3 and FLAG-H2B ChIP assays plotted against transcription rate of downstream genes (moving average, window 50). **(b) **Overlap between promoters upstream of active genes and the set of nucleosome-depleted promoters defined on the basis of depletion across the replicate H3 and FLAG-H2B experiments.

As previous studies show that *PHO5 *activation is accompanied by marked nucleosome loss in the promoter region [5,6], we sought to determine whether nucleosome depletion is a general attribute of active promoters. A total of 4,365 intergenic regions that reside immediately upstream of one or more validated yeast genes were assigned as promoters. Relative transcription rates were determined for each yeast gene from transcript levels measured by array and previously collected mRNA half-life data [16]. We found an inverse correlation of -0.39 between the enrichment of promoters in the H3 and H2B ChIP assays and the transcription rates of downstream genes (Figure 2a). Under the conditions examined, *PHO5 *is not induced and its promoter has an average nucleosome occupancy according to these datasets. To evaluate further the relationship between nucleosome depletion and transcription, we collated a set of 308 nucleosome-depleted promoters on the basis of their relative depletion across the replicate H3 and H2B experiments. Of these nucleosome-depleted promoters, 42% regulate highly active genes (Figure 2b). These data suggest that there is a systematic relationship between promoter strength and nucleosome depletion. However, as this correspondence is not complete there are likely to be other determinants of nucleosome occupancy.

### Transcription factor binding motifs are over-represented in nucleosome-depleted promoters

**Figure 3:**
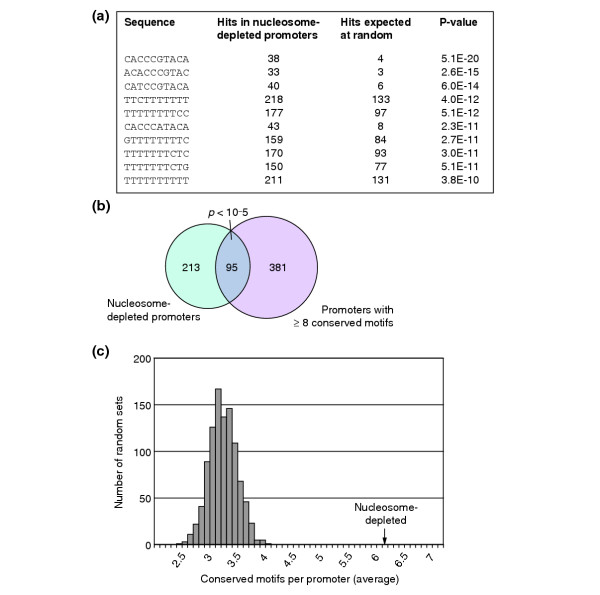
Sequence motifs over-represented in nucleosome-depleted promoters. **(a) **An unbiased search for sequences up to 10 bp in length over-represented in nucleosome-depleted promoters (relative to promoters overall) identified the poly(dA.dT) sequence element and variants of the Rap1 consensus motif ACACCCATACAT [21]. **(b) **Overlap between nucleosome-depleted promoters and promoters that contain multiple conserved motifs is shown [21]. **(c) **Histogram showing average numbers of motifs in 1,000 randomly generated promoter sets. Nucleosome-depleted promoters contain an average of 6.1 conserved motifs, significantly higher than in these randomly generated sets.

To identify additional determinants of occupancy, we sought sequence elements associated with nucleosome depletion. Specifically, we carried out an unbiased search for elements up to 10 bp in length that occur with higher frequency in nucleosome-depleted promoters. Two distinct categories of sequences emerged (Figure 3a). The first includes poly(dA.dT) elements. Stretches of 10 or more dA.dT nucleotides appear in 38% of depleted promoters, compared with 26% of promoters overall (hypergeometric *p *< 10^-5^). dA.dT stretches destabilize nucleosome formation *in vitro *and *in vivo *[17,18]. The enrichment of poly(dA.dT) elements in nucleosome-depleted promoters probably reflects, at least in part, this destabilizing influence. As a high proportion of the poly(dA.dT) elements identified in nucleosome-depleted promoters are more than 10 bp long (30% are at least 14 bp), these data do not address the minimum length required for destabilization. However, *in vitro *studies show that a 16-bp insertion leads to a 1.7-fold increase in accessibility of nucleosomal target sites [18].

The second sequence element enriched in nucleosome-depleted promoters corresponds to the consensus motif for the Rap1 transcription factor. This motif commonly occurs in the promoters of ribosomal proteins genes and is required for Rap1 binding *in vitro *and *in vivo *[19,20]. Some variant of this motif appears in 22% of nucleosome-depleted promoters, compared with just 8% of promoters overall (hypergeometric *p *< 10^-5^). Furthermore, multiple Rap1 sites are found in 19% of nucleosome-depleted promoters with Rap1 sites, compared to 8% of promoters with Rap1 sites overall (hypergeometric *p *< 10^-3^). These data suggest that Rap1 recruitment may lead to nucleosome loss.

Because only the Rap1 consensus site was identified in an unbiased search, we sought to identify additional sequence motifs by incorporating species conservation data. Specifically, we evaluated a set of 71 conserved motifs identified by Kellis and colleagues, a majority of which function in transcription factor recruitment [21]. Nearly half of these 71 motifs are over-represented in nucleosome-depleted promoters relative to promoters overall, as defined by a hypergeometric *p *< 0.001. However, many of the implicated motifs appear in the same promoters. For example, nine of the over-represented motifs are associated with filamentation gene promoters [21]. We therefore considered the possibility that the total number of conserved motifs might be a more relevant predictor of nucleosome depletion. Indeed, we found that 31% of nucleosome-depleted promoters contain at least eight motifs, compared with 11% of promoters overall (hypergeometric *p *<10^-5^; Figure 3b). Furthermore, nucleosome-depleted promoters contain an average of 6.1 motifs, whereas the average promoter contains 3.1 (permutation *p *< 0.001; Figure 3c). Next, we sought motifs associated with nucleosome depletion in the absence of multiple motifs, by confining our analysis to promoters containing a maximum of four motifs. This analysis identified just two over-represented motifs, which correspond to the Rap1 and Swi4 binding sites. Hence, although a large number of conserved motifs are enriched in nucleosome-depleted promoters, most appear to be relevant mainly when occurring in combination.

### Functionally cooperative transcription factors associate with nucleosome-depleted promoters

**Figure 4:**
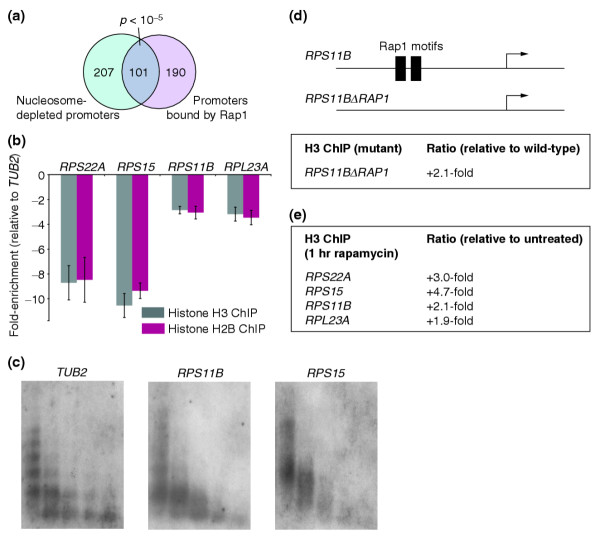
Nucleosome depletion in the vicinity of Rap1-binding sites. **(a) **Overlap between the 308 most nucleosome-depleted promoters and promoters found to recruit Rap1 in a global ChIP study [1]. **(b) **Nucleosome depletion in the vicinity of Rap1-binding sites in ribosomal gene promoters evaluated by ChIP. Fold-enrichment was determined by real-time PCR using primers that span Rap1-binding motifs in the *RPS22A*, *RPS15*, *RPS11B *and *RPL23A *promoters. **(c) **Southern blots showing DNA from yeast spheroplasts digested with increasing concentrations of micrococcal nuclease probed with labeled PCR products spanning the *TUB2 *promoter and the Rap1 sites in the *RPS11B *and *RPS15 *promoters. **(d) **Nucleosome occupancy for a mutant *RPS11B *promoter lacking Rap1 consensus sites was determined by H3 ChIP and real-time PCR. The mutant promoter is enriched 2.1-fold relative to wild type. **(e) **Nucleosome occupancy at Rap1-binding sites in ribosomal protein gene promoters after treatment with rapamycin evaluated by H3 ChIP and real-time PCR.

As a majority of the conserved motifs recruit transcription factors [21], we examined the relationship between transcription factor binding and nucleosome occupancy more directly. Lee and colleagues combined ChIP and microarrays to identify target promoters for essentially all yeast transcription factors under the same conditions used here to evaluate nucleosome occupancy [1]. For each factor, we determined the significance of overlap between its target promoters and the set of nucleosome-depleted promoters. Of the 113 transcription factors in their database, 31 tend to associate with nucleosome-depleted promoters as defined by a hypergeometric *p *< 0.001. Rap1 has the most significant association (Figure 4a), consistent with the enrichment of its binding motif (see above). Other top-ranked factors include Fhl1, which associates with many Rap1-bound promoters, and Swi4, whose binding motif is also enriched (Table 1).

We sought an underlying binding mechanism or function common to the transcription factors we had identified. However, these factors utilize a variety of binding domains, regulate different pathways, and only a minority have significant associations with promoters of highly active genes. Nonetheless, a commonality does emerge when transcription factor cooperativity is considered. A recent informatics study by Banerjee and Zhang identified 31 functionally cooperative transcription factor pairs (representing a total of 33 factors) on the basis of comprehensive binding and expression data [22]. Only a fraction of these are known to interact physically, suggesting that other mechanisms also confer cooperative function. There is a remarkable correspondence between these functionally cooperative factors and those that preferentially associate with nucleosome-depleted promoters (see Table 1). Of the 31 factors we found to associate with nucleosome-depleted promoters, 17 were found to be functionally cooperative by Banerjee and Zhang (*p *< 10^-5^). Furthermore, an evaluation of nucleosome occupancy at promoters bound by both members of a cooperative pair revealed a significant association with nucleosome-depletion for 18 of the 31 pairs (hypergeometric *p *< 0.01). Together, these findings suggest that binding motifs and transcription factors act in combination to deplete nucleosomes and suggest a role for nucleosomes in transcription factor cooperativity [23-25].

### Conditional nucleosome depletion at Rap1 consensus motifs

Although a number of transcription factors appear to act in defining promoter nucleosome occupancy, only the Rap1 consensus motif was identified in an unbiased search of nucleosome-depleted promoters. Furthermore, there is a highly significant association between nucleosome-depleted promoters and promoters bound by this factor *in vivo *[1] (Figure 4a). To investigate the relationship between Rap1 recruitment and nucleosome depletion further, we used ChIP and real-time PCR to evaluate nucleosome occupancy at several Rap1 binding sites in ribosomal protein promoters. We found that these regions are depleted 3- to 10-fold in H3 and FLAG-H2B ChIP assays, relative to a control promoter (*TUB2*) with average occupancy by global analysis (Figure 4b). We also used an orthogonal approach in which micrococcal nuclease digestion [26] was used to probe for nucleosomes at the *TUB2*, *RPS11B *and *RPS15 *promoters (Figure 4c). A pattern of nuclease protection indicative of a regular nucleosome array is evident at the *TUB2 *promoter, consistent with the average nucleosome occupancy attributed to this promoter by global ChIP analysis. In contrast, nuclease protection is not evident at the RAP1 sites in the *RPS15 *promoter, consistent with the marked nucleosome-depletion attributed to this region by global ChIP and real-time PCR analysis. The region surrounding the RAP1 sites in *RPS11B *exhibits weak nuclease protection, consistent with the modest nucleosome-depletion attributed to this region by global ChIP and real-time PCR. Although these focused analyses specifically addressed Rap1 sites in ribosomal protein genes, our global analyses indicate that approximately 30% of nucleosome-depleted promoters containing Rap1 motifs do not regulate ribosomal protein genes. Together these data confirm that nucleosomes are markedly depleted in the vicinity of Rap1 consensus sites *in vivo*, and thus extend previous studies showing that Rap1 induces local alterations in chromatin structure that, for example, result in increased nuclease sensitivity [27-29].

To gain further insight into the relationship between Rap1 and nucleosome depletion, we examined a mutant *RPS11B *promoter lacking its Rap1 consensus sites. We found that removal of these sites, which completely abrogates Rap1 binding [30], causes nucleosomes to return to the region, as reflected by a greater than twofold change in H3 ChIP enrichment (Figure 4d). We also examined the effect of rapamycin treatment on nucleosome occupancy in the vicinity of these consensus sites. Although ribosomal protein gene expression is dramatically reduced by rapamycin [31,32], Rap1 remains bound to its target promoters ([30,33], and B.B., E.P. and S.S., unpublished results). We found that rapamycin treatment causes nucleosomes to return to the vicinity of Rap1 sites, as reflected by twofold and greater increases in H3 ChIP enrichment (Figure 4e). Together these data show that Rap1 consensus sites are required for conditional nucleosome depletion at ribosomal protein gene promoters.

## Discussion

To gain further insight into the role of nucleosomes in gene regulation, we systematically evaluated promoter nucleosome occupancy in yeast by immunoprecipitating nucleosomal DNA and quantifying enrichment with microarrays. Promoters that are inefficiently immunoprecipitated by general anti-histone antibodies, and are therefore presumed to be relatively nucleosome-depleted, tend to regulate active genes (Figure 2). This is consistent with the previous observation that the activated *PHO5 *promoter is largely devoid of nucleosomes [5,6]. However, as not all nucleosome-depleted promoters regulate active genes, there are most likely to be additional determinants of depletion. An unbiased search for sequence elements enriched in nucleosome-depleted promoters revealed poly(dA.dT) elements, previously shown to destabilize nucleosome formation [17,18], and the Rap1 consensus motif. By incorporating sequence conservation data [21], more than 30 other enriched motifs could be identified. However, most of these appear to be relevant mainly when occurring in combination. When we limited this analysis to promoters containing four or fewer motifs, all but two of these additional motifs drop out (only the Rap1 and Swi4 consensus sites remain). As the majority of conserved motifs incorporated in this analysis recruit transcription factors [21], these data suggest that multiple transcription factors act in combination to deplete nucleosomes. This possibility is further supported by our finding that functionally cooperative transcription factors tend to bind nucleosome-depleted promoters. These associations may reflect a mechanistic model in which transcription factors compete collaboratively to displace nucleosomes in order to gain access to target sites in the DNA [23]. This model was formulated to explain why certain pairs of transcription factors bind cooperatively to proximal target sites *in vivo *and on a chromatin template, but not to naked DNA [23-25]. This view invokes a broad role for nucleosomes as ubiquitous negative regulators of transcription factor binding and function. We speculate that by promoting synergy among multiple transcription factors and impeding the activities of individual ones, nucleosomes facilitate threshold behavior and filter noise (for example, genetic variation in motif sequence) in the transcriptional regulatory network.

Although many factors appear to act in defining promoter nucleosome occupancy, our data indicate that Rap1 has a uniquely important role. Rap1 and its consensus motif are both markedly enriched in nucleosome-depleted promoters. Follow-up studies using real-time PCR and micrococcal nuclease digestion also demonstrate marked nucleosome depletion in the vicinity of Rap1 sites in the promoters of ribosomal protein genes. Moreover, nucleosomes appeared to return when the Rap1 consensus sites in one of these promoters were removed. These findings are consistent with previously described roles for Rap1 in opening chromatin and altering nucleosome positioning [27,28]. However, Rap1 recruitment is not equally associated with nucleosome depletion under all conditions. We find that nucleosomes partially return to the vicinity of Rap1 sites during a rapamycin-induced starvation response [34], even though Rap1 remains bound ([30,33], and B.B, E.P. and S.S., unpublished results). Hence, the nucleosome loss associated with Rap1 recruitment is most likely to require additional proteins, such as Esa1, a histone acetyltransferase recruited by Rap1 under exponential growth conditions but released in stress [30].

These findings may also offer insight into the barrier activity previously documented for Rap1 [35]. Heterochromatin propagation involves the sequential modification of histones in adjacent nucleosomes through positive-feedback mechanisms [7,11]. Certain factors such as Rap1 are able to block this propagation by largely unknown mechanisms [36]. One model speculates that these barriers create nucleosome-free 'holes' lacking the histone substrate required for heterochromatin propagation [29,35]. By identifying such a 'hole' in the vicinity of Rap1-binding sites *in vivo *our data support this model. Remarkably, the nucleosomal hole and the barrier function ascribed to Rap1 may be conditional, as nucleosomes return following treatment with the small molecule rapamycin, which activates a starvation response. Heterochromatic silencing has been shown previously to moderate under these conditions [37]. Hence, we speculate that dynamic influences on nucleosome occupancy may enable Rap1 to define chromatin domains and vary them in response to environmental cues.

More broadly, the widespread nucleosome loss observed in the promoters of active genes provides a general caveat for ChIP studies examining posttranslational histone modifications, as a decrease in signal for a histone modification at a promoter undergoing activation may actually reflect nucleosome loss. Similarly, regions that appear relatively hypo-modified by ChIP may actually be nucleosome-depleted. However, this is not the case for low levels of acetylation [38] and H3 lysine 4 methylation [39] observed at yeast telomeres, as these regions have high occupancy. The data also provide insight into the maintenance of epigenetic information by histone modifications. Whereas epigenetic memory of a repressed state can be maintained on histones in promoters, memory of an activated state must be maintained on histones outside the promoters, for example in transcribed regions, which may not undergo significant nucleosome loss during activation [5,6]. Methylation of histone H3 at lysines 4 and 36, targeted to transcribed regions in yeast via interactions between RNA polymerase and the methylases [39-47], may represent such 'activating' marks.

## Materials and methods

### Chromatin immunoprecipitation (ChIP)

DNA associated with histone H3 *in vivo *was immunoprecipitated with antibodies against the invariant H3 carboxy terminus using a ChIP protocol described previously [39,48,49]. Briefly, 45 ml log-phase w303a yeast (OD_600 _~ 1.0) growing in yeast extract/peptone/dextrose (YPD) were cross-linked in 1% formaldehyde for 15 min, washed twice in PBS, resuspended in 400 μl lysis buffer (50 mM Hepes-KOH pH 7.5, 140 mM NaCl, 1 mM EDTA, 1% Triton X-100, 0.1% sodium deoxycholate) and lysed with glass beads. The resulting extract was sonicated to fragment chromatin (4 × 20 sec burst/30 sec rest with a Branson Sonifier 250 at 70% duty, power 3) and centrifuged for 15 min. Solubilized chromatin was then immunoprecipitated with polyclonal antibodies against the carboxy terminus of histone H3 (Abcam or Cell Signaling). A unenriched whole-cell extract sample (WCE) was also retained as a control. After enrichment, cross-links were reversed by incubating samples in 10 mM Tris-HCl pH 8.0, 1 mM EDTA, 1.0% SDS, 150 mM NaCl at 65°C overnight. DNA was purified from ChIP and WCE samples by proteinase K treatment, phenol/chloroform extraction, ethanol precipitation, and incubation with RNAse. DNA associated with histone H2B *in vivo *was isolated in a similar manner from yeast containing epitope-tagged H2B [50] using anti-FLAG M2 monoclonal antibodies (Sigma).

### DNA amplification and hybridization

To obtain sufficient quantities for hybridization, immunoprecipitated DNA (from approximately 10^8 ^cells) and whole-cell extract DNA (unenriched control) were amplified in a linear fashion as described [51]. Briefly, terminal transferase was used to add poly(T) tails to DNA fragment and a T7-poly(A) adaptor primer was used to incorporate T7 promoters. The reaction products were used as template for an *in vitro *transcription reaction carried out with the T7 Megascript Kit (Ambion) and RNA samples were purified using an RNeasy Mini Kit (Qiagen). Amplified RNA was reverse-transcribed, incorporating amino-allyl dUTP, and the resulting DNA was fluorescently labeled by incubation with monofunctional reactive Cy5 (enriched sample) or Cy3 (unenriched control) dye as described [52]. Microarrays containing 6,438 PCR-amplified intergenic regions were prepared as described previously [39,53,54]. Mixed Cy5-/Cy3-labeled probe was hybridized to intergenic microarrays for 12-14 h at 60°C, washed and then scanned using a GenePix 4000A scanner with GenePix Pro software (Axon Instruments) as described [55]. In addition, transcript levels were determined by hybridizing Cy5-labeled mRNA extracted from log phase w303a yeast against Cy3-labeled genomic DNA on microarrays containing 6,218 open reading frames (ORFs), as described previously [16].

### Microarray data processing

Cy5 and Cy3 fluorescence were integrated for each feature using GenePix Pro Software (Axon). Data were processed and composite Cy5:Cy3 ratios determined according to protocols at the Stanford Microarray Database [56]. Correlations between replicate datasets were ~0.8 for all experiments. Composite datasets were log_2 _transformed and zero centered before further analysis. The histone H3 ChIP dataset was determined from four independent immunoprecipitations and hybridizations (two each using antibodies from Cell Signaling or Abcam). The FLAG-H2B ChIP dataset was determined from three independent immunoprecipitations and hybridizations. The mRNA dataset was determined from three independent extractions and hybridizations of mRNA against genomic DNA. Relative transcription rates were determined by dividing transcript levels by half-life data collected by Wang and colleagues [16]. A set of activated promoters was defined as those in the top 10% by mRNA expression level of associated gene, with divergent promoters assigned to the more highly expressed gene. Complete datasets are available online [57].

### Analysis of nucleosome-depleted promoters

Z-scores were assigned to each intergenic that reflect depletion across the four H3 and three H2B ChIP experiments, using the formula Z = (x - μ)/σ where x is the average of the replicate measurements, μ is the average of all intergenics and σ is the standard error of the replicate measurements. We defined as nucleosome-depleted the 410 features with the highest Z-scores. This set, which includes 308 promoters, contains nucleosome-depleted outliers and is not inclusive of all promoters that immunoprecipitate with average or lower efficiency. The average aqueous enrichment ratio [15] for these 308 depleted promoters is 1.7-fold, significantly higher than expected by chance (permutation *p *< 0.001), consistent with the premise that these promoters are relatively free of nucleosomes.

Sequence elements common to nucleosome-depleted promoters were identified by searching between 10 and 500 bp upstream of gene start sites for over-represented sequences up to 10 bp in length using the GeneSpring program suite (Silicon Genetics). Enrichment was confirmed by evaluating the significance of overlap between the set of nucleosome-depleted promoters and the set of promoters containing Rap1 consensus motifs (ACACCCATACAT with up to two mismatches) or poly dA.dT stretches at least 10 bp in length (identified using PatMatch, *Saccharomyces *Genome Database [58]). Statistical significances of overlaps between sets are expressed as *P*-values calculated by a hypergeometric probability model. The *P*-values reflect the extent to which observed overlaps exceed that expected under the null hypothesis that there is no relationship between the sets [59]. Where specified, permutation analyses were carried out by generating 1,000 random but representative promoter sets with an Excel macro and used to confirm statistical significance. Lists of promoters containing the 71 conserved motifs [21] were collated from gene sets available online [60]. Lists of promoters bound by transcription factors at a significance of *p *< 0.001 [1] were collated from data available at [61].

### Real-time PCR

Regions approximately 200 bp in size that span one or more Rap1 consensus sites in ribosomal protein gene promoters were amplified from ChIP and unenriched control samples using SYBR green PCR mix (Qiagen) in an MJ Research real-time PCR machine according to the manufacturers' instructions. Fold-ratios that reflect relative enrichment or depletion of a given region in the H3 or FLAG-H2B ChIP assays were determined using the 2^-ΔΔC^_T _method described in the Applied Biosystems User Bulletin. For each region examined, the *TUB2 *promoter was used as the normalizer (this promoter is used as a control because its occupancy approximates that of the average promoter by global analysis), and the unenriched control sample was used as the calibrator. Each reported ratio represents the average of three independent ChIP experiments analyzed in duplicate by real-time PCR. The following primer pairs were used:

*RPS22A promoter: *5'-GCCTAAAACGCCCATAAGTT-3' and 5'-ACTGCAAACCCATATTCAAGA-3'

*RPS15 promoter: *5'-TACACCGCGCGTATAAATCA-3' and 5'-CCCAGCAAGGAGTTTCTCAG-3'

*RPS11B promoter: *5'-GAAGAAATATTTCCTTGCTGCACC-3' and 5'-AAGGGAAACGTAAAGCTATTGGAC-3'

*RPL23A promoter: *5'-ATTAACATCTGTACACCCCCAACT-3' and 5'-TACAGTTCGTTTCCTGCC ATATTA-3'

*TUB2 promoter: *5'-GGCCTAACAGTAAAGATATCCTCC-3' and 5'-GTTGTAGTAGCTGCTATGT CACTC-3'

Centromeric vectors containing either a mutant *RPS11B *promoter lacking the two Rap1 consensus motifs [30] or an essentially wild-type allele were transformed into wild-type yeast and used in an H3 ChIP assay to evaluate the consequence of removing Rap1 binding sites on nucleosome occupancy. Enrichment was evaluated by real-time PCR using the following primer pair that selectively amplifies the plasmid alleles but not the endogenous *RPS11B *promoter: 5'-CTGGAAGAAATATTTCCTT GCTCTAG-3' and 5'-AAGGGAAACGTAAAGCTATTGGAC-3'.

### Micrococcal nuclease assay

Log-phase cultures of W303a yeast grown in 450 ml YPD to OD_600 _of 1.0 were spheroplasted with zymolase (10 mg in 40 ml volume of 1 M sorbitol, 50 mM Tris pH 7.4, 10 mM β-mercaptoethanol (β-ME), at 30°C for 38 min shaking at 300 rpm), divided into five aliquots, and digested with increasing concentrations (20 U to 320 U) of micrococcal nuclease (Worthington Biochem) in 600 μl 0.5 mM spermidine, 1 mM β-ME, 0.075% NP-40. DNA from digested samples was extracted with phenol twice and chloroform once and precipitated in ethanol. Samples were washed, resuspended in 10 mM Tris pH 7.5, subjected to RNAse treatment, cleaned up with the MinElute kit (Qiagen) and run out in a 1% agarose gel. Following depurination, denaturation and neutralization of the gel, DNA was transferred onto nylon membranes by capillary action and covalently linked to the membranes by UV irradiation. Southern blotting was carried out using a DIG Luminescent Detection Kit (Roche) and DIG-labeled probe generated by PCR using the *TUB2*, *RPS11B *and *RPS15 *primers described above.

## Figures and Tables

**Table 1:** Transcription factors that tend to associate with nucleosome-depleted promoters

Transcription factor	Pathway	Number of targets	Nucleosome-depleted	Functionally cooperative
Rap1	Biosynthesis	291	35%	
Fhl1	Biosynthesis	137	48%	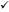
Swi4	Cell cycle	165	36%	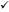
Hsf1	Environmental response	114	35%	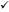
Gat3	Metabolism	119	31%	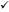
Cin5	Environmental response	200	23%	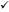
Phd1	Metabolism	138	25%	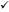
Dal81	Metabolism	70	34%	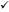
Ndd1	Cell cycle	122	26%	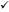
Yap6	Environmental response	123	26%	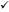
Fkh2	Cell cycle	145	24%	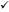
Pdr1	Environmental response	103	27%	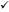
Ino4	Metabolism	118	25%	
Smp1	Environmental response	99	27%	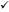
Yap5	Environmental response	113	26%	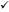
Ash1	Development	41	41%	

Transcription factors are ranked according to the significance of their association with nucleosome-depleted promoters, as determined by a hypergeometric model. Shown are the 16 top-ranked factors along with relevant physiologic pathway, number of promoters bound [1], and percent of target promoters that are nucleosome-depleted. Factors found previously to be functionally cooperative are indicated [22].
